# Intra-operative neuromonitoring and electrophysiological nerve stimulation for the spinal accessory and marginal mandibular nerve during neck dissection: a scoping review

**DOI:** 10.1017/S0022215126105064

**Published:** 2026-08

**Authors:** Aditya Singh, James Heyman, David Owens

**Affiliations:** 1 Cardiff University School of Medicine, UK; 2 Department of Otolaryngology, Cardiff and Vale University Health Board, UK

**Keywords:** Intra-operative neurophysiological monitoring, Accessory nerve, Neck dissection

## Abstract

**Objectives:**

Evaluating the effectiveness of intra-operative nerve monitoring and stimulation in preventing spinal accessory nerve and marginal mandibular nerve injuries during neck dissection.

**Methods:**

Preferred Reporting Items for Systematic Reviews and Meta-Analyses Extension for Scoping Review methodology was used to map and summarise current literature. Online PubMed/MEDLINE searches and manual screening identified relevant studies for inclusion.

**Results:**

Seven studies met the inclusion criteria. Six addressed the spinal accessory nerve and one the marginal mandibular nerve. No randomised controlled trials were identified. The evidence tentatively supports the use of intra-operative neurophysiological monitoring in reducing spinal accessory nerve and marginal mandibular nerve palsy rates during neck dissection. Intra-operative neurophysiological monitoring modalities varied across studies, from continuous neuromonitoring to handheld nerve stimulation and nerve mapping. Monitoring criteria and outcome definitions differed across studies.

**Conclusions:**

The evidence base for intra-operative neurophysiological monitoring of standard accessory nerve and marginal mandibular nerve during neck dissection remains methodologically heterogeneous. Standardised protocols, prospective controlled studies and dedicated marginal mandibular nerve monitoring studies are required before routine implementation can be recommended.

## Introduction

Operating in the head and neck carries the risk of cranial nerve injury. The marginal mandibular nerve (MMN) and spinal accessory nerve (SAN) are among the most vulnerable structures during routine benign, oncological and reconstructive operations.^1^

The MMN is a terminal branch of the facial nerve (cranial nerve VII). It innervates muscles of the lower lip, particularly the depressor anguli oris and depressor labii inferioris. It typically crosses the facial artery near the inferior border of the mandible but may descend up to 1 to 2 cm below the mandibular angle, placing it at high risk of manipulation and damage during operative procedures.^2^ In addition to parotidectomy, submandibular gland excision and neck dissection, level I–III cervical node resections, facial reconstructive surgery, mandibular osteotomies, rhytidectomy and cutaneous lesion excisions in the submandibular or upper neck region all pose a risk to the MMN.^3^^–^^5^ Injury to the MMN can result in complications such as lower lip aesthetic asymmetry, impaired articulation, oral incompetence and aesthetic disfigurement.^6^

The SAN innervates the sternocleidomastoid (SCM) and trapezius muscles. It enters the SCM from its deep surface and exits through the posterior border. It then traverses level V of the neck, running superficial to the levator scapulae. The SAN enters the trapezius from its deep surface.^7^ It is susceptible to damage during elective and therapeutic neck dissection procedures, resection of level V lesions and open biopsies of cervical lymphadenopathy.^8^^,^^9^ The nerve’s superficial course through level V exposes it during flap elevation or nodal clearance. Nerve injury during such procedures may go unnoticed if not actively monitored, causing complications like shoulder ptosis, scapular winging and neuropathic pain (a collection of symptoms often termed shoulder syndrome).^7^^,^^8^

Historically, surgeons have often relied on anatomical landmarks, experience and tactile cues to identify these nerves during surgery. The MMN is commonly approximated by its relationship to the angle of the mandible. The Hayes-Martin manoeuvre, described in 1951, represents a systematic approach to MMN preservation. This step involves ligating the posterior facial vein and superiorly reflecting the investing fascia below the mandible, thereby protecting the nerve within the fascial envelope.^10^^,^^11^ The SAN is often located by identifying its presence at Erb’s point, in relation to the tendon of the SCM, or by palpating the transverse process of the C2 vertebra. However, these methods lack precision and may be inadequate in cases of aberrant anatomy or extensive disease.^7^^,^^12^

Recent advances in intra-operative neurophysiological monitoring (IONM) include the use of continuous electromyographic (EMG) monitoring, threshold-based trend analysis and integration with surgical navigation systems. Emerging technologies such as near-infrared spectroscopy, ultrasound-aided nerve identification and enhanced imaging modalities have the capability to develop more effective nerve preservation methods further.^13^^,^^14^

This study aims to map and characterise the existing evidence base for IONM of the MMN and SAN during neck dissection.

## Methodology

This scoping review was conducted in accordance with the Preferred Reporting Items for Systematic Reviews and Meta-Analyses Extension for Scoping Reviews (PRISMA ScR guidelines).^15^ Eligible studies were identified based on adult patients (≥18 years) undergoing selective, modified or radical neck dissections with use of IONM targeting the SAN or MMN. Only studies reporting clinical or functional outcomes (e.g. EMG, shoulder or facial nerve function, patient-reported metrics) were included. Eligible designs included randomised controlled trials (RCTs), controlled clinical trials, cohort studies and retrospective observational studies. Studies published after 2006 were included.

Studies were excluded if they involved animal- or cadaver-based methodologies, cohort sizes less than 10, or if they did not include findings specific to the SAN or MMN.

Articles were searched across PubMed/MEDLINE on 25 June 2025 using the following prespecified search strategy:

*(‘intra-operative nerve monitoring’[Title/Abstract] OR ‘IONM’[Title/Abstract] OR ‘nerve integrity monitoring’[Title/Abstract] OR ‘NIM’[Title/Abstract] OR ‘intra-operative neuromonitoring’[Title/Abstract] OR ‘real-time nerve monitoring’[Title/Abstract] OR ‘electromyographic monitoring’[Title/Abstract] OR ‘EMG monitoring’[Title/Abstract] OR ‘nerve stimulation’[Title/Abstract]) AND (‘neck dissection’[Title/Abstract] OR ‘cervical lymphadenectomy’[Title/Abstract] OR ‘radical neck dissection’[Title/Abstract] OR ‘modified radical neck dissection’[Title/Abstract] OR ‘selective neck dissection’[Title/Abstract] OR ‘functional neck dissection’[Title/Abstract] OR ‘lymph node dissection’[Title/Abstract]) AND (‘marginal mandibular nerve’[Title/Abstract] OR ‘MMN’[Title/Abstract] OR ‘facial nerve branch’[Title/Abstract] OR ‘mandibular branch’[Title/Abstract] OR ‘spinal accessory nerve’[Title/Abstract] OR ‘SAN’[Title/Abstract] OR ‘accessory nerve’[Title/Abstract]) AND (‘nerve injury’[Title/Abstract] OR ‘iatrogenic injury’[Title/Abstract] OR ‘nerve palsy’[Title/Abstract] OR ‘nerve damage’[Title/Abstract] OR ‘neurapraxia’[Title/Abstract] OR ‘axonotmesis’[Title/Abstract] OR ‘neurotmesis’[Title/Abstract] OR ‘shoulder dysfunction’[Title/Abstract] OR ‘shoulder syndrome’[Title/Abstract]).*

Alongside database searches, grey literature was systematically searched using predefined search strategies, including targeted website searches, Google Scholar queries and citation reference list screening of included studies. Eligible records were identified through these methods and manually uploaded and screened for inclusion and/or exclusion.

**Figure 1.**
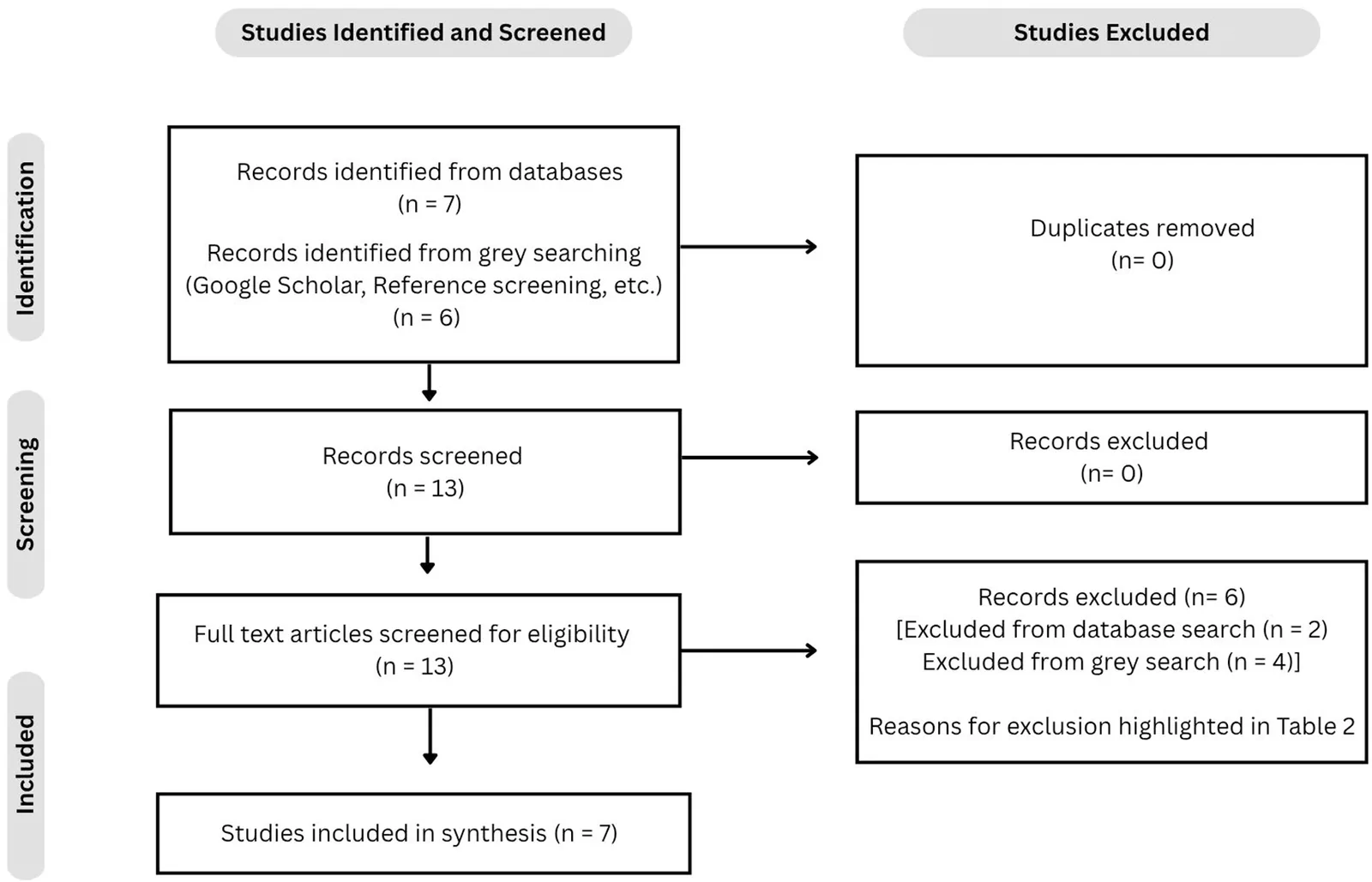
PRISMA framework of studies screened, reviewed and included in our analysis. Figure 1 long description.

Characteristics of included and excluded studies are summarised in Tables 1 and 2. Figure 1 illustrates the number of studies reviewed and included or excluded in our analysis according to the PRISMA ScR Framework. The Newcastle-Ottawa Scores were used to calculate the risk-bias of each study. These were used to appraise non-randomised studies across selection, comparability and outcome domains (Table 3).

**Table 1.** Characteristics of included studies (PICOS framework) Table 1 long description.

Authors	Population	Nerve studied & intervention	Comparator	Outcomes	Study design	Key findings
Lee *et al*., 2013^16^	25 male patients with head and neck cancer undergoing SND/MRND.	SAN; IONM (Medtronic NIM−2.0).	None.	Shoulder function (shrug, flexion, abduction, winging), pain score, EMG (fibrillations, PSWs); assessed at 1 month.	Prospective single-arm cohort.	No gross movement deficits: 88% patients had shoulder pain (mean pain score = 2.3 ± 1.3); EMG fibrillations 40%, PSWs 64%; pain score correlated with EMG changes (*p* < 0.005). Patients with more EMG evidence of denervation tended to have higher pain scores, despite intact shoulder function.
Birinci *et al*., 2014^17^	17 patients, 20 neck sites studied (14 SND, 3 MRND).	SAN; IONM: significant change defined by threshold/amplitude criteria using NIM-Response 2.0 Nerve Integrity Monitor (Medtronic).	Significant vs insignificant amplitude changes during IONM.	EMG at 1 month; CMS & GARS scores pre-operatively, 1 and 2 months.	Prospective double-blinded clinical trial.	EMG at one month showed 5% normal, 30% mild, 50% partial, and 15% total axonal degeneration. CMS and GARS both worsened post-op, with less CMS deterioration and GARS recovery by month 2 only in shoulders with insignificant IONM changes.
Svenberg Lind *et al*., 2015^19^	18 patients undergoing unilateral neck dissections.	SAN; IONM mapping (NIM−3); SAN vs cervical plexus stimulation.	SAN vs cervical plexus as trapezius innervation.	CMAP amplitudes intra-op; post-op shoulder function check.	Prospective intra-operative mapping study.	SAN innervated all trapezius regions; cervical plexus contributed motor input (∼40−43% CMAP) in 39% of patients. SAN loss of signal occurred in 5/18 cases, all developing next-day trapezius paresis.
Tirelli *et al*., 2018^11^	71 patients undergoing neck dissections (36 NIM, 35 non-NIM).	MMN; NIM-Response 2.0 (Medtronic).	Non-IONM vs IONM-assisted groups.	MMN function (House-Brackmann Grading) evaluated on day 1 post-op, on discharge, and regularly on follow-up for ongoing palsy.	Prospective comparative cohort study.	NIM significantly reduced nerve injury rates: total palsy 4.08% vs 20.93% in controls (*p* = 0.021) and permanent palsy 0% vs 11.63% (*p* = 0.019). No significant increase in operative time was observed between groups.
Cossa *et al*., 2023^20^	64 patients undergoing FRND (31 non-IONM, 33 IONM).	SAN; NIM-Response® 3.0 System (Medtronic).	Non-IONM vs IONM-assisted SAN identification.	CMS; EMG when deficit; permanent palsy defined at 6 months.	Retrospective comparative cohort study.	SAN lesions: 22.6% without IONM (12.9% permanent) vs 6.1% with IONM (0% permanent); permanent reduction significant (*p* = 0.0495).
Ling *et al*., 2024^21^	17 patients enrolled, 14 with complete participation.	SAN; NIM-Neuro 3.0 stimulator (Medtronic).	Early shoulder dysfunction vs none.	Constant Score & NDII at pre-op, 72h, 1 month, 6 months; US & SWE.	Prospective single-arm cohort.	Higher pre-closure threshold at 1 mA in dysfunction group (*p* = 0.034); worse Constant Score at 6 months (*p* = 0.024); US/SWE not discriminative.
Polistena *et al*., 2021^28^	56 MRND cases for thyroid cancer	SAN; NIM-Response 3.0 system (Medtronic).	Non-IONM vs IONM-assisted SAN identification.	SPADI at day 3 & 6 months; EMG if symptomatic.	Retrospective observational cohort study.	IONM during lateral neck dissection reduced temporary and permanent SAN injury rates compared with visualisation alone, though differences were not statistically significant. Overall SAN lesion rate was 10.7%.

CMS = Constant-Murley score; CMAP = compound muscle action potential; EMG = electromyography; GARS = General Activity Restriction Score; IONM **=** intra-operative neuromonitoring; MMN **=** marginal mandibular nerve; MRND = modified radical neck dissection; NIM = nerve integrity monitor; PSW = positive sharp waves; SAN = spinal accessory nerve; SND = selective neck dissection; SPADI = shoulder pain and disability index; SWE = shear wave elastography; US = ultrasound.

**Table 2.** Characteristics of excluded studies Table 2 long description.

Study	Reason for Exclusion
Eastwood, M. J. & George, A. P., 2018^22^	Cadaver study; no patient outcomes reported.
Sood *et al*., 2015^23^	Facial nerve focus; not SAN or MMN.
Savvas *et al*., 2016^24^	Observational study; facial nerve; not relevant to RQ.
McGarvey *et al*., 2014^25^	Review; SAN/MMN but not original clinical data.
Palacios-García *et al*., 2021^26^	Description of IONM technique, with some SAN discussion; lacks data on SAN injury rates.
Eisele *et al*., 1988^27^	Study more than 20 years old.

MMN = marginal mandibular nerve; RQ = research question; SAN = spinal accessory nerve.

**Table 3.** Newcastle-Ottawa scores for included studies Table 3 long description.

Study	Selection	Comparability	Outcomes	Total
Lee *et al*., 2013	3	0	2	5/9
Birinci *et al*., 2014	4	1	2	7/9
Svenberg Lind *et al*., 2015	2	0	2	4/9
Tirelli *et al*., 2018	4	1	3	8/9
Cossa *et al*., 2023	4	1	3	8/9
Ling *et al*., 2024	3	0	3	6/9
Polistena *et al*., 2021	4	1	3	8/9

## Results

Following database searches, the inclusion and exclusion criteria were employed to select seven studies in this analysis and exclude six. Of the included studies, six addressed the SAN, and one focused on the MMN. Across the SAN literature, IONM modalities included continuous EMG monitoring, handheld intra-operative nerve stimulation with threshold measurements and intra-operative nerve mapping. The single MMN study only reported findings with a handheld nerve stimulator during neck dissection.

### Spinal accessory nerve outcomes

The impact of IONM on SAN injury and recovery is multi-faceted. A single-arm prospective cohort study (*n* = 25) published by Lee *et al*. found that intra-operative SAN monitoring in selective neck dissection patients resulted in no significant post-operative shoulder syndrome after 1 month, although 88 per cent reported mild shoulder pain (average pain score, 2.3 ± 1.3).^16^ EMG at 1 month showed fibrillation in 40 per cent and positive sharp waves in 64 per cent of the cohort, indicating subclinical axonal injury despite good clinical function. These cases correlated with patients experiencing shoulder pain.

Birinci *et al*. reported a prospective, double-blinded cohort study investigating 20 neck sites with predetermined ‘significant IONM change’ as a threshold increment greater than or equal to 0.25 mA and/or amplitude decrement greater than or equal to 72 per cent.^17^ Shoulders with insignificant IONM signals intra-operatively showed less deterioration and faster recovery of Constant-Murley Scores (CMS) and activity restriction scores (*p* <0.05). The CMS is a validated 100-point composite shoulder function tool evaluating pain, activities of daily living, range of motion and strength.^18^ EMG abnormalities at 1 month remained common, with 50 per cent partial axonal degeneration and 15 per cent experiencing total paralysis.

Svenberg Lind *et al*. conducted an intra-operative mapping study (*n* = 18).^19^ Trapezius innervation was mapped, and the study reported a loss of SAN signal in 5/18 cases at the end of the surgery, which was followed by next-day trapezius paresis. Two of these patients had shoulder weakness despite intact innervation from the cervical plexus. This supports the potential relationship between intra-operative electrophysiological changes and early post-operative motor impairment in certain patients.

Ling *et al*.’s prospective cohort study (*n* = 25) found an association between high pre-closure stimulation thresholds and early shoulder dysfunction with worse CMS values at six months post-surgery.^21^ Radiographic findings such as ultrasound and shear wave measures were not discriminative in this small cohort.

Polistena *et al*. retrospectively analysed 56 patients undergoing modified radical neck dissection (MRND).^28^ They reported that 89.29 per cent had no shoulder deficits, with 8.93 per cent showing temporary impairment and 1.79 per cent with permanent deficits. However, the use of IONM was non-random and increased over time, reducing the interpretability of results. The authors note that IONM use was subject to factors such as progressive standardisation, surgeons’ learning curves, equipment availability and technical issues. This introduces a potential time-trend and selection confounding when comparing IONM vs non-IONM groups. The study also reported a non-significant difference between the IONM and non-IONM group outcomes.

Cossa *et al*. (2023) conducted a retrospective comparative study of 64 patients undergoing functional radical neck dissection (FRND) for thyroid cancer metastases.^20^ The non-IONM group had an SAN lesion rate of 22.6 per cent, with 12.9 per cent of the cohort experiencing permanent deficits. The IONM group had a 6.1 per cent SAN lesion rate with no permanent deficits. Narrow inclusion and exclusion criteria in their study (including reoperations, prior neck radiotherapy and metastatic nodes > 3 cm) potentially introduce homogeneity within the cohort.

A systematic review by McGarvey *et al*. found it unclear whether IONM consistently reduces SAN injury or predicts functional outcomes, citing limited and contradictory evidence.^25^ The *Annals of Thyroid* review by Li *et al*. noted a lack of standardised protocols and prognostic criteria for SAN IONM.^29^ These discrepancies may stem from methodological variations and differing definitions of ‘significant change’ in IONM parameters.

### Marginal mandibular nerve outcomes

Tirelli *et al*. prospectively analysed 71 patients, of whom 36 had IONM-assisted neck dissections and 35 non-IONM patients.^11^ This study reported lower MMN palsy rates in the IONM-assisted group, with a total palsy rate of 4.08 per cent and no cases of permanent damage. The non-IONM group reported a palsy rate of 20.93 per cent and 11.63 per cent cases with permanent damage. The differences between these groups were statistically significant (*p* = 0.021). IONM implementation added no additional operative time and limited additional financial costs.

## Discussion

Neck dissection is associated with a substantial burden of SAN and MMN injuries, with prevalence varying by the extent of the procedure and anatomy dissected. A systematic review by Houlind Larsen *et al*. reported a high prevalence of shoulder syndrome following radical neck dissection, while also highlighting that MMN injury rates are reported less often and may be under-ascertained.^30^ This background supports ongoing interest in adjuncts such as IONM to improve operative safety. However, as this scoping review demonstrates, the literature is characterised by methodological heterogeneity, small sample sizes, variable monitoring protocols and inconsistent outcome definitions. These characteristics limit quantitative synthesis and instead highlight an evidence base that remains largely at an early stage of development.

The most immediate observation from this review is that the volume of clinical literature specifically addressing IONM for the SAN and MMN during neck dissection is limited. Seven studies met the inclusion criteria in total, spanning a period of two decades. No RCTs were identified for either nerve. The predominant study designs were small prospective cohort studies and retrospective comparative analyses, with sample sizes in primary clinical studies ranging from 17 to 71 patients. Two previous reviews by McGarvey et *al*. (2014) and Li *et al*. (2019) reached similar conclusions regarding the limited evidence base at their respective time points.^25^^,^^29^ This present review also finds that the methodological landscape has not substantially changed in these years. Despite the clinical significance of SAN and MMN injuries during neck dissection, the field has not yet produced the volume or quality of evidence needed to characterise its role with confidence.

A defining characteristic of the included literature is the variability in what is meant by IONM across studies. Intra-operative neurophysiological monitoring modalities range from continuous EMG recordings as described by Lee *et al*.^16^ to threshold-based handheld stimulation at two defined time points as shown by Birinci *et al*. and Ling *et al*.^17^^,^^21^ Svenberg Lind *et al*. employed a distinct approach, mapping the relative contributions of the SAN and cervical plexus branches to trapezius innervation using compound muscle action potentials at supramaximal stimulation intensity.^19^ Cossa *et al*. combined an initial mapping phase with endpoint stimulation,^20^ whilst Polistena *et al*. used a handheld probe with stimulation at initial identification and again at the end of dissection.^28^ Tirelli *et al*. conducted the sole MMN study, which employed continuous EMG recording from electrodes placed in the depressor muscles alongside a handheld probe.^11^ These represent genuinely distinct approaches.

However, what is consistently absent across the literature is not simply the distinction between continuous recording and handheld stimulation, but rather clarity about which component of the monitoring system is driving the reported outcomes. In most nerve integrity monitor (NIM) systems, continuous EMG recording and handheld nerve stimulation coexist. Yet, no included studies specify the relative contribution of each modality to intra-operative decision-making or the outcome.

Furthermore, the criteria used to define a clinically meaningful electrophysiological change vary substantially. Birinci *et al*. defined significant change as a threshold increment of greater than or equal to 0.25 mA and/or amplitude decrement of greater than or equal to 72 per cent.^17^ Cossa *et al*. used amplitude loss greater than 50 per cent and/or latency increase greater than 10 per cent.^20^ Ling *et al*. focused on pre-closure stimulation threshold at 1 mA as a prognostic parameter.^21^ Svenberg Lind *et al*. used complete loss of signal.^19^ Polistena *et al*. described significant changes in M-wave amplitude, waveform or any threshold increase on the nerve monitor.^28^ No two studies applied the same criteria. Li *et al*. (2019) explicitly identified this variability in monitoring criteria as a fundamental barrier to meaningful synthesis,^29^ and the present review confirms that it remains unresolved. Without consensus on modality, parameters and definitions of significant change, the findings of individual studies cannot be reliably compared or aggregated.

An additional concern is that repeated electrical nerve stimulation has itself been identified as a potential cause of neurapraxia.^31^ The frequency, amplitude and timing of handheld stimulation during each case are not reported in any of the included studies, meaning that the monitoring intervention itself carries a risk that most literature neither accounts for nor quantifies.

Svenberg Lind *et al*. found that cervical plexus branches provide approximately 40 per cent of neural input to all 3 functional parts of the trapezius in 39 per cent of patients, which is a magnitude comparable to that of the SAN.^19^ Notably from the study, only two out of five patients with an intra-operative loss of SAN signal had an intact cervical plexus, but both still developed trapezius paresis. This suggests that cervical plexus contribution may be more relevant to long-term reinnervation than to short-term function following SAN injury. It also implies that monitoring the SAN alone may be insufficient to characterise the risk to trapezius function. This anatomical caveat represents an underexplored domain of surgical technique and IONM strategy. Future studies should consider whether monitoring the cervical plexus motor branches during neck dissection requires attention alongside the SAN.

Across the included studies, the outcomes used to assess nerve function are heterogeneous. These outcome measures ranged from functional scores, such as the Shoulder Pain and Disability Index (SPADI) and CMS, to more objective modalities such as EMG and ultrasonography. Lee *et al*. reported that no patients developed shoulder syndrome, yet 40 per cent had fibrillation potentials and 64 per cent had positive sharp waves on EMG, indicating subclinical axon injury.^16^ If examination findings were relied upon solely, the extent of nerve injury in this cohort would have appeared negligible. Similarly, Polistena *et al*. conducted EMG only in patients with abnormal clinical scores. This could mean that patients with subclinical injury but normal SPADI scores did not undergo EMG evaluation, potentially underestimating the true injury burden in their series. If the field is to generate standardised and comparable evidence, a consensus on a core outcome set incorporating clinical, EMG and patient-reported measures is prudent.

Of all the included studies, only one addressed the MMN. This is disproportionate given the clinical significance of MMN injury, which carries significant functional and aesthetic consequences.^6^ Houlind *et al*. also highlighted the under-ascertainment of MMN injury rates in broader neck dissection literature.^30^ If MMN outcomes are not routinely reported in neck dissection series, the apparent clinical burden of MMN injury can remain underestimated, reducing the perceived imperative for MMN neuromonitoring research.

The comparative studies included in this review provide useful contextual observations but might not be representative of the general adoption of IONM in neck dissections due to heterogeneity in the literature. Polistena *et al*. reported an increasing adoption of IONM with time, alongside growing surgical experience and technical refinement.^28^ This introduces a potential secular trend confounding which can weaken the study’s generalisability. Cossa *et al*. controlled for surgeon variability by including a single high-volume surgeon’s data, which can again reduce the representativeness of the sample.^20^ Across other studies, surgeon experience was not accounted for despite its known effect on outcomes, representing an important confounding factor.

A limitation of this review is that the search was conducted on PubMed/MEDLINE with manual supplementation. Relevant studies indexed in other databases or in grey literature may therefore not have been included. Hence, publication bias cannot be excluded. Secondly, the included evidence was small and methodologically heterogeneous. This limits comparability and precludes quantitative synthesis.

## Conclusion

This scoping review identifies a limited and methodologically heterogeneous evidence base for IONM of the SAN and MMN during neck dissection. Despite over a decade of literature, no RCTs exist, monitoring protocols remain non-standardised, and outcome definitions vary considerably between studies. The MMN remains critically under-represented. The current evidence is insufficient to draw firm conclusions about IONM’s efficacy but collectively suggests a potential benefit which warrants rigorous investigation. Priorities for future research include the development of a standardised IONM protocol, prospective controlled trials, agreed core outcome sets and dedicated MMN monitoring studies. Cost-effectiveness evaluation is imperative before any recommendation for routine IONM implementation.

## Figure 1 Long description

The flowchart outlines the process of study identification, screening and inclusion. It begins with ′Identification,′ where records are identified from databases (7 records) and grey searching (6 records). The next step is ′Screening,′ where 13 records are screened. Following this, full-text articles are screened for eligibility, totaling 13 articles. The final step is ′Included,′ where 7 studies are included in synthesis. On the right side, ′Studies Excluded′ are shown, with 0 duplicates removed and 0 records excluded initially. Further exclusion includes 6 records, with 2 excluded from database search and 4 from grey search. Reasons for exclusion are highlighted in Table 2.

Navigate back to Figure 1.

## Table 1 Long description

Study characteristics and findings are summarized for seven head and neck surgery studies assessing intra-operative nerve monitoring during neck dissection and related procedures. Most studies focused on the spinal accessory nerve and postoperative shoulder outcomes, using measures such as shoulder function tests, pain scores, electromyography changes, and shoulder disability scales; one study evaluated the marginal mandibular nerve. Comparative cohorts reported fewer nerve injuries with monitoring than without it, including lower total and permanent palsy rates for the marginal mandibular nerve and fewer spinal accessory nerve lesions with fewer permanent deficits. Single-arm and mapping studies reported that postoperative pain could track with electromyography signs of denervation even when gross shoulder movement appeared intact, and that loss of intra-operative signal was followed by early trapezius weakness. One trial linked smaller intra-operative amplitude changes to better recovery on shoulder scores by two months. Ultrasound and shear wave elastography did not clearly distinguish early dysfunction in one small cohort. Interpretation is limited by mixed designs, small samples, varied comparators, and different outcome timing across studies.

Navigate back to Table 1.

## Table 2 Long description

Excluded studies are listed with the specific reason each did not meet the review criteria. Eastwood and George was excluded because it was a cadaver study without patient outcomes. Sood et al. and Savvas et al. were excluded for focusing on the facial nerve rather than the target nerves and for not addressing the research question. McGarvey et al. was excluded because it was a review and did not provide original clinical data. Palacios-García et al. was excluded because it mainly described intraoperative nerve monitoring technique and did not report spinal accessory nerve injury rates. Eisele et al. was excluded because it was more than 20 years old. Overall, exclusions were driven by study type, relevance to the target nerves and question, missing outcome data, and publication age.

Navigate back to Table 2.

## Table 3 Long description

Newcastle-Ottawa quality assessment scores are listed for seven included studies across selection, comparability, and outcomes domains, with an overall total out of nine. Total scores range from four out of nine to eight out of nine. Three studies scored eight out of nine: Tirelli 2018, Polistena 2021, and Cossa 2023; Birinci 2014 scored seven out of nine. Mid-range totals include Ling 2024 with six out of nine and Lee 2013 with five out of nine, while Svenberg Lind 2015 had the lowest total at four out of nine. Selection scores vary from two to four, comparability is either zero or one, and outcomes range from two to three. Comparability is the most consistently low domain, with four studies receiving zero. Scores summarize study quality by this scale and do not by themselves indicate effect size or clinical importance.

Navigate back to Table 3.
